# Profile of Orthodontic Use across Demographics

**DOI:** 10.3390/dj11120291

**Published:** 2023-12-15

**Authors:** Man Hung, Golnoush Zakeri, Sharon Su, Amir Mohajeri

**Affiliations:** 1 College of Dental Medicine, Roseman University of Health Sciences, 10894 S. River Front Parkway, South Jordan, UT 84095, USA; gzakeri879@student.roseman.edu (G.Z.);; 2 Division of Public Health, University of Utah, Salt Lake City, UT 84108, USA; 3 School of Dentistry, University of the Pacific, San Francisco, CA 94103, USA

**Keywords:** dentistry, orthodontics, braces, retainers

## Abstract

Objectives: Population-based studies have focused on patients’ rendering of orthodontic treatment due to their malocclusion and medical needs. However, there is a scarcity of data from national sources on the prevalence of orthodontic visits and associated patient characteristics, as well as their effect on dental health. This study evaluated the demographic differences of orthodontic patients and examined the association between orthodontics use and risk of periodontal disease and oral surgical intervention. Methods: This study used data collected from the 2019 Medical Expenditure Panel Survey. Descriptive statistics were used to evaluate socio-demographics and covariates by the use of orthodontics. Chi-square tests were used to assess demographic differences among respondents who stated whether or not they used orthodontics. Logistic regression models were then used to examine the association of orthodontics and oral health outcomes. Results: The sample size was 12,422, of which 491 respondents indicated a usage of orthodontics. There were significant differences among demographic variables (*p* < 0.05) that included 61.1% females, 60.8% Whites, 67.6% participants under the age of 18 years old, and a family total income of $100,000 or more (52.7%). After controlling for socio-demographics and covariates, individuals who used orthodontics were less likely to have periodontal disease (AOR = 0.623, 95% CI = 0.610 to 0.637) and tooth extractions (AOR = 0.071, 95% CI = 0.070 to 0.073) than those who did not. Conclusions: Our findings indicate orthodontics usage was higher in females, younger patients, and Whites, highlighting the possible demographic disparities in orthodontics use. Additionally, those who used orthodontics were less likely to have oral health issues.

## 1. Introduction

Orthodontics is a specialized branch of dentistry that is dedicated to the diagnosis, prevention, and management of misalignments in the teeth and jaws, often referred to as craniofacial anomalies and malocclusion. These discrepancies from the ideal occlusion, or perfect alignment, can vary widely in both type and severity, which in turn dictates the nature and length of the orthodontic treatment required [1].

The spectrum of orthodontic issues encompasses a wide range of conditions—from simple cases like minor crowding or spacing of teeth to complex scenarios involving severe jaw misalignments. Treatment strategies are equally varied, including the use of braces, clear aligners, retainers, and sometimes even orthognathic surgery, and the approach is tailored to the individual patient’s needs. If these orthodontic issues are not addressed, they can have far-reaching effects. Pronounced misalignments can lead to significant challenges in maintaining oral hygiene, potentially resulting in tooth decay, gum disease, and even tooth loss. Difficulty with chewing and speech, as well as the progression of jaw discomfort or temporomandibular joint disorders, are possible physical consequences [2]. Moreover, there can be psychosocial impacts; for instance, severe dental misalignments may affect a person’s self-esteem and social interactions.

In the landscape of dental care, a significant and notable trend has been the marked increase in individuals pursuing orthodontic treatments in recent years. This surge is closely associated with a transformation in the demographic composition of orthodontic patients, especially with an impressive escalation in adult patients seeking such treatments [3]. The data from a span of two years, between 2016 and 2018, revealed that approximately 60,000 adults in the (U.S.) took steps to correct their dental alignment through orthodontic procedures [4]. This notable trend underscores a shift in societal attitudes toward orthodontics, as well as the acknowledgment of the enduring benefits of such treatments, regardless of the patient’s age. The growing influx of adult orthodontic patients emphasizes the significance of a deeper understanding of their demographic profiles. It brings to light the varying needs and preferences that differ markedly from those of the younger cohort traditionally associated with orthodontic care. Adults may seek treatment for functional reasons, such as improving bite and jaw alignment, but aesthetic motivations are equally compelling, driving demand for innovative and less noticeable treatment modalities. This demographic shift towards older patients in orthodontics suggests the need for adaptations in treatment access, availability, and preferences. Adults may have more complex dental needs due to restorations, missing teeth, or other periodontal issues, necessitating more customized treatment plans. Furthermore, many adult patients look for discrete treatment options that fit into professional and social lifestyles, such as clear aligners, ceramic braces, or behind-the-teeth (lingual) braces.

Understanding the nuances of this demographic shift is crucial for orthodontic practice and planning. It can guide orthodontists in tailoring their services to meet the unique needs of a more mature population, inform decisions about practice offerings, and influence the development of policies that increase access to care for all age groups. It can also spur innovation in treatment technologies that cater to adult preferences and lifestyles.

Previous research efforts have been directed at discerning orthodontic treatment preferences among various demographic segments, taking into consideration a range of factors including gender, age, and socio-economic status [5]. These studies have revealed a striking disparity in the utilization of orthodontic services, particularly when it comes to children from minority groups. Specifically, Hispanic and African American communities have been shown to have a lower likelihood of pursuing orthodontic care, despite the presence of clinically recognized needs [6]. Among these groups, the African American population, in particular, has been noted to have the lowest propensity for seeking out such treatments compared to other demographics [7].

Additionally, gender appears to play a significant role in the pursuit of orthodontic treatment. Females are generally more proactive about seeking orthodontic care than males, which may reflect broader societal trends regarding beauty and self-image [7]. This gender-based difference in healthcare utilization underlines the need for increased awareness and perhaps tailored approaches to encourage a more balanced uptake of orthodontic care across genders.

Despite the observed demographic disparities, the impact of malocclusion—the misalignment of teeth and jaws—is a universal concern. Studies suggest that about 15% of the adult population may experience negative social perceptions and functional difficulties as a result of malocclusion [8]. Moreover, a considerable segment of the population, ranging from 57% to 59% across different racial backgrounds, exhibits varying levels of need for orthodontic intervention [8]. This suggests that malocclusion is a widespread issue that transcends racial and ethnic boundaries, although access to and utilization of orthodontic treatment may not be equally distributed.

Numerous studies have delved into how orthodontic treatments may affect the broader spectrum of oral health-related quality of life, but the exploration into their direct impact on overall oral health has been less extensive [9]. There is complexity in establishing clear-cut conclusions, with some research indicating a neutral correlation—implying that while orthodontic procedures do not pose substantial risks to periodontal health, they also do not offer significant periodontal benefits [7]. Additionally, a systematic review concluded that orthodontic treatment does not prevent future periodontal problems, except for specific unusual malocclusions [10]. This suggests that the primary impacts of orthodontic treatment may be more closely related to alignment and occlusal factors rather than periodontal health directly. However, contrasting with these neutral findings, other systematic review found that orthodontic treatment appears to have a transient impact on the subgingival microbiome composition, while it does not seem to significantly alter the salivary microbiome, thus maintaining a consistent level of microbial diversity [11]. Research has also shed light on the positive side effects of orthodontic interventions, particularly emphasizing improvements in oral hygiene for individuals who have undergone such treatments [12]. These studies advocate that straightening teeth can lead to better accessibility for cleaning, thereby reducing the risks of plaque accumulation and gingivitis, which are precursors to more serious periodontal diseases. The discourse around orthodontics and periodontal health is further enriched by considering the fact that well-aligned teeth are easier to maintain and less likely to harbor food particles and bacteria, which can lead to decay and gum disease. As such, the long-term maintenance of oral health may be facilitated by the ease of cleaning that comes with properly aligned teeth.

Despite the wealth of research on orthodontic trends and preferences, recent nationwide data, especially those highlighting the frequency of orthodontic visits and their associated patient demographics, are somewhat limited. Given the rising trajectory of orthodontic treatments, it becomes imperative to revisit and reanalyze demographic data to pinpoint and address existing disparities. Moreover, a discernible void exists in current literature regarding the direct effects of orthodontic practices on comprehensive oral health. Consequently, this study aimed to examine the demographic profile of individuals opting for orthodontic treatments, with a particular focus on orthodontics on oral health.

## 2. Materials and Methods

### 2.1. Data Source

The data utilized in this study were derived from the consolidated file of the 2019 Medical Expenditure Panel Survey (MEPS) [13]. Conducted by the Agency for Healthcare Research and Quality (AHRQ), MEPS is a rich source of data on healthcare costs, utilization, and insurance coverage in the U.S. To ensure ethical integrity, the MEPS obtained necessary approvals from institutional review boards or ethics committees, following standard research protocols to protect participants’ rights and privacy.

The study setup for MEPS 2019 involved a complex, stratified, multistage probability design, which is detailed in AHRQ’s MEPS survey background [14] and elsewhere [15]. This design was aimed at producing nationally representative data. Informed consent was a critical aspect of the survey, ensuring that all participants were fully aware of the study’s purpose, their voluntary participation, and the confidentiality of their responses.

MEPS used a combination of questionnaires, including computer-assisted personal interviewing and computer-assisted telephone interviewing [14]. The questionnaires were subjected to rigorous testing and validation processes to ensure their reliability and accuracy in capturing healthcare-related data. The inclusion criteria for MEPS are generally broad, aiming to reflect a wide demographic cross-section of the U.S. civilian non-institutionalized population. Conversely, the survey has specific exclusion criteria, such as excluding individuals residing in long-term care facilities. Before participation, individuals were briefed about the survey’s scope, the nature of the questions, and the time commitment required. This pre-briefing helped in managing participant expectations and improving the quality of responses. Trained interviewers then administered the questionnaires and provided necessary clarifications. 

MEPS is a comprehensive survey designed to gather information about healthcare and healthcare expenditures among individuals in the U.S. It seeks to offer insights into healthcare for various population groups, both on a national and regional scale, across different time periods. One of the key aspects of MEPS is its dedication to ensuring that the conclusions drawn from the survey data accurately represent the broader U.S. population. To achieve this, MEPS employs a statistical method known as “weighting”. This process involves assigning different weights to survey responses based on how the data were collected. Essentially, it ensures that the findings can be generalized to the entire U.S. population, accounting for the diversity and variations in the survey’s sample groups [16,17].

### 2.2. Measures

#### 2.2.1. Socio-Demographic Variables

The age of individuals was divided into five groups: under 18 years old, 18–24 years old, 25–44 years old, 45–64 years old, and 65+ years old. Race/ethnicity was grouped into five categories of non-Hispanic White, non-Hispanic Black, non-Hispanic Asian, non-Hispanic other-race or multiple-race, and Hispanic. The gender consisted of males and females. Annual household income was divided into five categories: less than $10,000, $10,000–$24,999, $25,000–$49,999, $50,000–$99,999, and $100,000 or more. The region was categorized as Northeast, Midwest, South, and West. Education was categorized into 8 groups: no degree, GED, high school diploma, bachelor’s degree, master’s degree, Doctorate degree, other degree, and under 16—inapplicable. The acronym “GED” stands for “General Educational Development.” It refers to a set of tests that provide individuals who have not completed their high school education in the U.S. with an opportunity to earn the equivalent of a high school diploma. The GED tests assess knowledge and skills in core subject areas such as language arts (reading and writing), mathematics, science, and social studies. Health insurance was categorized into three categories of private, public, and uninsured, and dental insurance was a binary variable of yes and no.

#### 2.2.2. Dependent Variables

Outcome measures included the need for periodontal and surgical treatment: (1) Gum disease: have you ever had periodontal scaling, root planing, or gum surgery? and (2) Tooth extraction: have you ever had an extraction, tooth pulled, or other oral surgery?

#### 2.2.3. Independent Variable

The independent variable representing the use of orthodontics was the following question: have you used any orthodontia, braces, or retainers? (yes/no).

#### 2.2.4. Covariates

Risk factors associated with poor oral health included tobacco use and diabetes history. The diabetes variable was dichotomous (Yes/No): have you ever been diagnosed with diabetes? The tobacco use variable was “How often do you smoke cigarettes?”, and it was converted to a binary variable with the responses collapsed into Yes and No by grouping the Every Day and Some Days responses under Yes and Not at All responses under No.

#### 2.2.5. Statistical Analyses

Descriptive statistics of participants’ socio-demographics and covariates by the use of orthodontics were calculated. Chi-square tests were used to assess demographic differences among respondents who stated whether or not they used orthodontics. Binary logistic regression models were fit to evaluate the associations between orthodontics use and oral health outcomes such as gum disease and tooth extraction after adjustment for sociodemographic characteristics and covariates that might influence oral health, thereby isolating the effect of orthodontic treatment. The results were presented as adjusted odds ratios (AORs) with corresponding 95% confidence intervals (CIs). AORs offer a measure of the strength and direction of the association between orthodontic appliance use and oral health outcomes, after accounting for other variables. In assessing the results, a two-sided *p*-value of less than 0.05 was considered statistically significant. This threshold indicated that the observed associations were unlikely to be due to chance alone and could be deemed reliable within the standard conventions of statistical analysis. The complex sampling design of the MEPS dataset was taken into account by applying sampling weight [18] in regression models and sample demographic estimates to generalize the findings to the U.S. population.

## 3. Results

The MEPS serves as an extensive data source for healthcare research, including studies on orthodontic treatment and its effects on population health. For the year under consideration, the survey engaged 44,339 participants, a substantial sample intended to reflect a wide cross-section of the U.S. population. From this cohort, a proportion of data was missing for the outcome measures, with 16,090 participants having incomplete information regarding the oral health outcomes of interest. Furthermore, an additional 15,827 respondents had missing data for the sampling weight variable, which is critical for ensuring that the analysis accounts for the complex survey design and is representative of the larger population. After these exclusions, the final analytical sample comprised 12,422 respondents—a subset of the original group but still sizable for robust statistical analysis. Within this final sample, 491 individuals reported the use of orthodontic devices—namely orthodontia, braces, or retainers. 

The MEPS survey employs a design that ensures its results are reflective of the broader U.S. populace. The survey’s methodology and weighting procedures allow for generalizations from the sample to the entire country’s population. In this case, the weighted data from the 12,422 respondents are projected to represent an estimated 149,823,417 people living in the U.S. in the year 2019. Moreover, the subset of 491 respondents who indicated the use of orthodontic devices extrapolates to an estimated 6,229,933 individuals in the U.S. population. 

Table 1 shows the demographics for individuals with and without orthodontic retainers. Of the 491 participants who self-reported orthodontics, 67.6% were under the age of 18. Non-Hispanic Whites (60.8%) and females (61.1%) made up the majority of those who claimed they had orthodontics. 

Many people had private health insurance (77.3%) and dental coverage (58.8%). Furthermore, most (52.7%) of those reporting orthodontic use had a family total income of $100,000 or more (Table 2).

The majority had no history of diabetes (99.0%) or tobacco use (94.9%). Most of those with orthodontics were from the Southern part (37.5%) of the U.S. (Table 3). Table 1, Table 2 and Table 3 provide a detailed comparison of demographic characteristics, as well as the history of diabetes or tobacco use between individuals who used orthodontics and those who did not. 

Approximately 0.8% of the participants had gum disease, and 3.9% had tooth extraction. After controlling for socio-demographics, diabetes, and tobacco use, individuals who used orthodontics were less likely to have gum disease (AOR = 0.623, 95% CI = 0.610 to 0.637, *p* < 0.05) and tooth extraction (AOR = 0.071, 95% CI = 0.070 to 0.073, *p* < 0.05) than those who did not use.

## 4. Discussion

### 4.1. Syntheses

Leveraging the 2019 MEPS, this research underscores that determinants beyond medical rationale influence the adoption of orthodontics [13]. A notable observation was that individuals with a history of orthodontic engagement generally exhibited superior oral health attitudes [19]. Undergoing orthodontic treatments can spur increased dental visits, in turn promoting heightened oral health consciousness and preventive practices [20]. The regular appointments necessary for orthodontic care provide repeated interactions with dental professionals, offering opportunities for patient education on oral hygiene importance [21,22]. The visibility and physical presence of orthodontic devices may serve as a constant reminder of the investment in one’s oral health, motivating individuals to maintain their teeth and gum condition [23]. This includes routine practices like brushing and flossing. 

A salient data point was the 67.6% of the population under 18 years old reporting orthodontic use, reflecting a cultural normal of embracing these treatments early in life, possibly due to increased value placed on dental alignment and aesthetics during formative years. This age group’s engagement suggests a broader societal wellness perspective, where orthodontic treatment is a part of personal and social development. Gendered distinctions were evident, with 61.1% of females reporting orthodontic use, possibly due to societal pressures targeting women’s physical appearance [24]. Women, facing intense societal scrutiny, may be more likely to seek treatments to align their physical image with aesthetic ideals [25]. 

Ethnicity emerged as a significant factor; treatments were predominantly favored by non-Hispanic Whites. This trend might signal issues such as differential access to care, varying health literacy levels, or distinct cultural attitudes towards orthodontic treatments among ethnic groups. Socioeconomic status, intertwined with ethnicity, critically influenced the quest for aesthetic enhancements and orthodontic interventions [25]. For patients under 18 years old, parental income was pivotal in dictating the realization of orthodontic treatments, overriding individual preferences. In nations marked by pronounced income disparities, like the U.S., those in higher economic strata were more probable recipients of orthodontic care [26].

Recognizing the demographic leanings in orthodontic treatment aids in discerning disparities in accessibility and affordability. These insights arm policymakers and healthcare practitioners to craft strategies, ensuring care permeates across socioeconomic layers. Additionally, it aids in gauging treatment impacts on oral health outcomes and socio-cultural implications, potentially guiding holistic patient care [27].

After adjusting for confounding variables such as demographics, the presence of diabetes, and tobacco consumption, a significant association between orthodontic treatment and enhanced oral health outcomes was observed. Orthodontic interventions showed a protective effect against gum disease [28] and a reduced propensity for tooth extraction [20], emphasizing the potential benefits beyond cosmetic enhancements. These treatments, by fostering optimal dental alignment, promote effective oral hygiene practices, mitigating risks associated with dental diseases [20].

Overall, this study highlights a multifaceted decision-making landscape for individuals considering orthodontic treatment, extending beyond clinical and medical necessity. Social norms, cultural values, demographic characteristics, and broader health implications significantly influence the adoption of orthodontic interventions [29]; conditions and individual cases may vary. 

### 4.2. Strengths and Limitations

In interpreting the results of this study, it is important to acknowledge its inherent limitations. Primarily, the reliance on self-reported data for oral health outcomes poses challenges, as such data can be vulnerable to recall inaccuracies and other potential biases, possibly skewing the findings. Another constraint pertains to the structure of the MEPS itself. The limited range of MEPS response options might not capture the nuanced experiences of the respondents. Offering a more granular set of response options might have yielded richer insights. A third notable limitation is that the study has less power to establish causal links between the use of orthodontics and oral health outcomes. This limitation stems from the cross-sectional nature of the data, which captures a snapshot in time rather than tracking changes over a prolonged period. Additionally, crucial data elements such as reasons for extraction and treatment alternatives were not available, limiting the possibility of conducting a more comprehensive analysis of oral health.

Despite these constraints, a significant strength of our study is the utilization of a vast, nationally representative sample. This approach ensures that our findings are not just isolated observations but resonate with a larger, overarching narrative relevant to the entire U.S. population. This broad representation is invaluable in research, ensuring that results are not merely idiosyncratic or confined to specific sub-groups but reflect a wider national trend. The sheer scale and diversity of this sample provide robustness to our findings, even as we remain cautious about potential biases. Moreover, by highlighting these disparities and gaps in our current understanding, this study effectively sets the stage for future investigations. Future research, informed by our findings, can focus more incisively on the nuances of orthodontic treatment adoption across various demographics, thereby advancing the comprehension of this critical aspect of oral healthcare.

## 5. Conclusions

This research indicated that individuals receiving orthodontic treatments may experience fewer oral health complications. However, it is important to consider that these results might not solely be due to orthodontic procedures. Often, those who can afford orthodontic treatments also have greater financial resources, which could contribute to better overall oral health. Furthermore, the adoption of orthodontic interventions was notably prevalent among females and non-Hispanic Whites, in addition to those in higher income brackets. Analyzing orthodontic treatment across varied demographics offers critical perspectives on treatment accessibility, oral health implications, and broader societal well-being. These insights have the potential to guide policy formulation, refine treatment methodologies, and improve the dental care journey for all.

## Figures and Tables

**Table 1:** Demographics of participants who used orthodontics versus who those did not.

	Total (Sample = 12,422; Population = 149,823,417)	Individuals with Orthodontia, Braces, or Retainers (Sample = 491; Population = 6,229,933)	Individuals without Orthodontia, Braces, or Retainers (Sample = 11,931; Population = 143,593,484)	*p*-Value
	weighted *n* (%)	weighted *n* (%)	weighted *n* (%)	
Age				
Less than 18 years old	38,731,797 (25.9)	4,209,911 (67.6)	34,521,887 (24.0)	
18 to 24 years old	11,777,489 (7.9)	1,046,313 (16.8)	10,731,175 (7.5)	
25 to 44 years old	31,980,467 (21.3)	593,149 (9.5)	31,387,318 (21.9)	<0.05
45 to 64 years old	38,758,721 (25.9)	308,378 (4.9)	38,450,343 (26.8)	
65 or more years old	28,574,942 (19.0)	72,181 (1.2)	285,002,761 (19.8)	
Gender				
Male	68,476,744 (45.7)	2,421,805 (38.9)	66,054,940(46.0)	<0.05
Female	81,346,672 (54.3)	3,808,128 (61.1)	77,538,544 (54.0)	
Race/Ethnicity				
Hispanic	20,548,024 (13.7)	1,237,425 (19.9)	19,310,599 (13.4)	
Non-Hispanic White	102,380,515 (68.3)	3,785,355 (60.8)	98,595,160 (68.7)	
Non-Hispanic Black	13,554,858 (9.0)	723,178 (11.5)	12,831,680 (8.9)	<0.05
Non-Hispanic Asian	8,566,854 (5.8)	254,046 (4.1)	8,312,807 (5.8)	
Non-Hispanic other race	4,773,166 (3.2)	229,929 (3.7)	4,543,238 (3.2)	
Education				
No degree	9,731,365 (6.5)	768,349 (12.3)	8,963,016 (6.3)	
GED	2,478,772 (1.7)	22,009 (0.4)	2,456,763 (1.7)	
High school diploma	38,830,936 (26.0)	843,134 (13.5)	37,987,802 (26.5)	
Bachelor’s degree	29,566,943 (19.8)	417,548 (6.7)	29,149,395 (20.2)	<0.05
Master’s degree	16,579,729 (11.1)	157,951(2.5)	16,421,778 (11.5)	
Doctorate degree	3,753,278 (2.4)	11,950 (0.2)	3,741,328 (2.6)	
Other degree	12,220,898 (8.2)	109,698 (1.8)	12,111,200 (8.5)	
Under 16—Inapplicable	36,376,094 (24.3)	3,899,294 (62.6)	32,476,800 (22.7)	

**Table 2:** Income and health insurance coverage of participants who used orthodontics versus those who did not.

	Total (Sample = 12,422; Population = 149,823,417)	Individuals with Orthodontia, Braces, or Retainers (sample = 491; Population = 6,229,933)	Individuals without Orthodontia, Braces, or Retainers (Sample = 11,931; Population = 143,593,484)	*p*-Value
	weighted *n* (%)	weighted *n* (%)	weighted *n* (%)	
Family’s total income				
Less than $10,000	5,172,245 (3.5)	158,671 (2.5)	5,013,574 (3.5)	
$10,000 to $24,999	11,838,803 (7.9)	349,851 (5.6)	11,488,951 (8.0)	
$25,000 to $49,999	24,207,452 (16.1)	659,258 (10.6)	23,548,194 (16.4)	<0.05
$50,000 to $99,999	43,800,814 (29.2)	1,779,134 (28.6)	42,021,680 (29.3)	
$100,000 or more	64,804,103 (43.3)	3,283,018 (52.7)	61,521,084 (42.8)	
Health insurance				
Any private	113,387,008 (75.7)	4,814,390 (77.3)	108,572,619 (75.6)	
Public only	32,899,093 (22.0)	1,203,243 (19.3)	31,695,849 (22.1)	<0.05
No insurance	3,537,316 (2.3)	212,300 (3.4)	3,325,016 (2.3)	
Dental insurance				
No	68,622,604 (45.9)	2,569,426 (41.2)	66,053,179 (46.1)	<0.05
Yes	80,867,236 (54.1)	3,660,507 (58.8)	77,206,729 (53.9)	

**Table 3:** Lifestyle factors of participants who used orthodontia, braces, or retainers and those who did not.

	Total (Sample = 12,422; Population = 149,823,417)	Individuals with Orthodontia, Braces, or Retainers (Sample = 491; Population = 6,229,933)	Individuals without Orthodontia, Braces, or Retainers (Sample = 11,931; Population = 143,593,484)	*p*-Value
	weighted *n* (%)	weighted *n* (%)	weighted *n* (%)	
Region				
Northeast	26,912,037 (18.1)	1,231,703 (19.8)	25,680,334 (17.9)	
Midwest	33,252,111 (22.2)	1,138,864 (18.3)	32,113,248 (22.4)	<0.05
South	51,919,355 (34.7)	2,338,396 (37.5)	49,580,959 (34.6)	
West	37,406,337 (25)	1,520,969 (24.4)	35,885,367 (25.1)	
Diabetes history				
No	138,386,492 (92.4)	6,152,221 (99.0)	132,234,271 (92.1)	<0.05
Yes	11,394,955 (7.6)	59,110 (1.0)	11,335,845 (7.9)	
Tobacco history				
No	101,295,896 (91.4)	1,920,897 (94.9)	99,374,999 (91.4)	<0.05
Yes	9,500,790 (8.6)	102,906 (5.1)	9,397,884 (8.6)	

## Data Availability

The data for this study are freely available at https://meps.ahrq.gov/data_stats/download_data_files.jsp (accessed on 17 November 2023).
